# Prevalence of asymptomatic malaria and bed net ownership and use in Bhutan, 2013: a country earmarked for malaria elimination

**DOI:** 10.1186/1475-2875-13-352

**Published:** 2014-09-04

**Authors:** Kinley Wangdi, Michelle L Gatton, Gerard C Kelly, Archie CA Clements

**Affiliations:** Research School of Population Health, College of Medicine, Biology and Environment, The Australian National University, Canberra, ACT Australia; University of Queensland, Infectious Disease Epidemiology Unit, School of Population Health, Brisbane, Queensland Australia; Phuentsholing General Hospital, Phuentsholing, Bhutan; School of Public Health & Social Work, Queensland University of Technology, Brisbane, Queensland Australia

**Keywords:** Malaria, Long-lasting insecticidal bet nets, Bhutan, Asymptomatic malaria

## Abstract

**Background:**

With dwindling malaria cases in Bhutan in recent years, the government of Bhutan has made plans for malaria elimination by 2016. This study aimed to determine coverage, use and ownership of LLINs, as well as the prevalence of asymptomatic malaria at a single time-point, in four sub-districts of Bhutan.

**Methods:**

A cross-sectional study was carried out in August 2013. Structured questionnaires were administered to a single respondent in each household (HH) in four sub-districts. Four members from 25 HH, randomly selected from each sub-district, were tested using rapid diagnostic tests (RDT) for asymptomatic *Plasmodium falciparum* and *Plasmodium vivax infection.* Multivariable logistic regression models were used to identify factors associated with LLIN use and maintenance.

**Results:**

All blood samples from 380 participants tested negative for *Plasmodium* infections. A total of 1,223 HH (92.5% of total HH) were surveyed for LLIN coverage and use. Coverage of LLINs was 99.0% (1,203/1,223 HH). Factors associated with decreased odds of sleeping under a LLIN included: washing LLINs <six months and >nine months compared to washing LLINs every six months; HH in the least poor compared to the most poor socio-economic quintile; a HH income of Nu 5,001-10,000 (US$1 = Nu 59.55), and Nu >10,000, compared to HH with income of <Nu 1,500; HH located one to three hours walking distance to a health centre compared to being located closer to a health centre; a reported lack of knowledge as to what to do in event of LLINs being torn; and keeping LLINs in a box compared to keeping them hanging in the place of use. Factors associated with use of LLINs for purposes other than the intended use included: income group Nu 1,501-3,000 and HH located one to three hours walking distance from a health centre.

**Conclusions:**

There was high coverage of LLINs in the study area with regular use of LLINs throughout the year. LLIN use for purposes other than malaria prevention was low. With high coverage and regular use of LLINs, and a zero prevalence of malaria infection found in historically high-risk communities during the peak malaria season, it appears Bhutan is on course to achieve malaria elimination.

## Background

Malaria remains one of the most important infectious diseases globally, with an annual incidence of 300–500 million cases and nearly one million deaths per year, imposing an enormous burden of suffering in tropical regions of the world [1, 2]. However, there has been an estimated 17% global reduction of malaria incidence from 2000–2009 [3, 4]. This improvement has been made possible by a substantial increase in investment in tackling malaria globally, in addition to rapid economic development and urbanization in many endemic countries. The scaling up of interventions has reduced malaria burden and transmission in many endemic areas [5–7]. Today, of the 99 malaria-endemic countries, 32 are pursuing an elimination strategy and 67 are controlling malaria [2, 8, 9]. The World Health Organization (WHO) Southeast Asia region (SEAR) has seen a particularly rapid reduction in malaria in the last decade [10].

**Figure 1:**
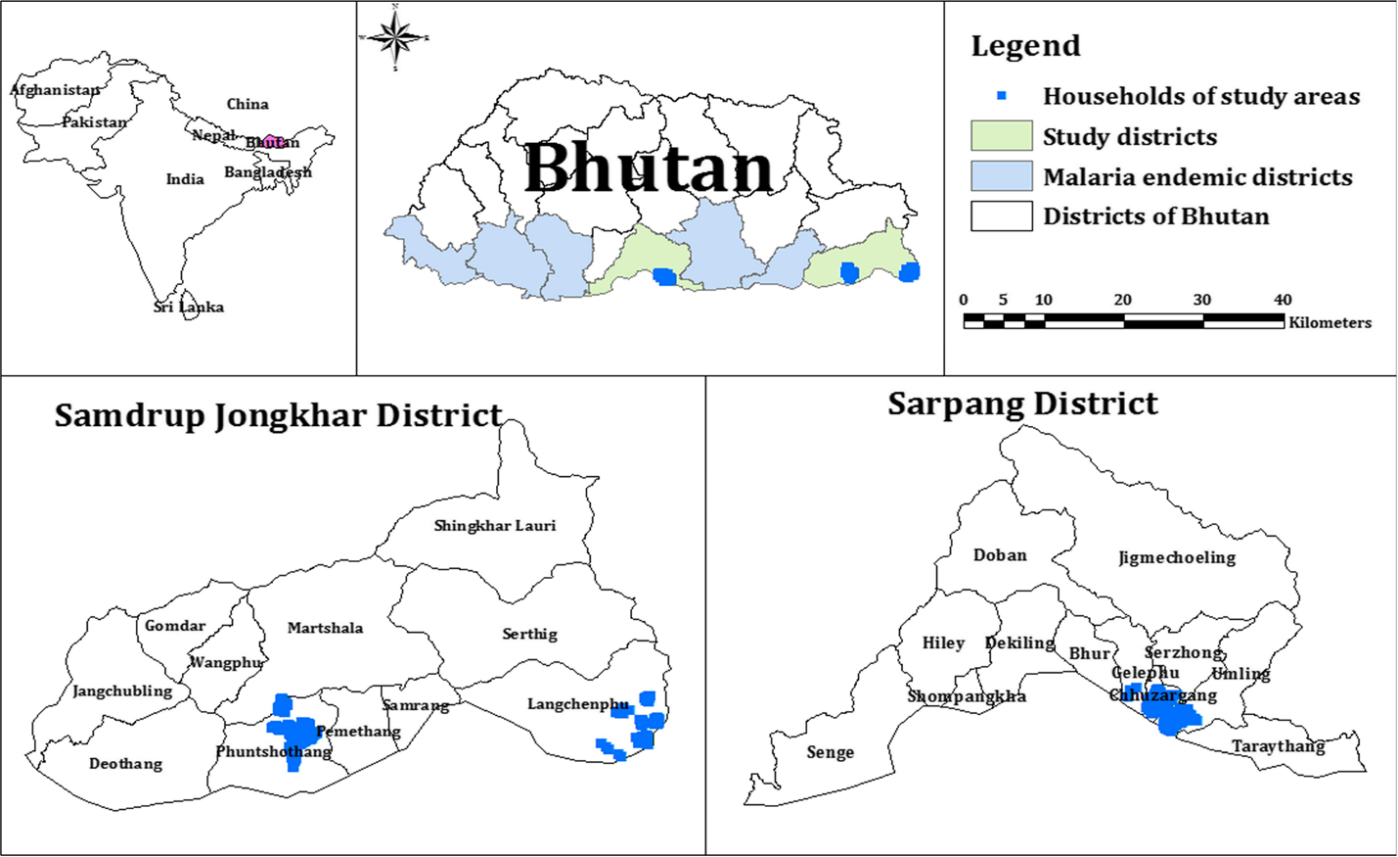
**Malaria endemic districts and study area.**

Numbers of malaria cases have been dwindling in Bhutan in recent years. As a result, Bhutan announced a national strategy to eliminate malaria by 2016 [11]. Malaria is usually reported in seven districts in the southern belt of Bhutan, bordering India (Figure 1) [12]. The population at risk of malaria in these seven districts was 309,662 in 2013, including, by district: Chukha 85,608, Dagana 26,553, Pemagatshel 24,646, Samdrup Jongkhar 39,405, Samtse 68,579, Sarpang 43,915, and Zhemgang 20,956 [13]. These districts border the Indian states of Assam and West Bengal, which report among the highest numbers of cases of malaria by state in India [14–17]. In these border areas, the climate is sub-tropical with abundant rainfall in the summer months, providing an environment that is conducive for multiplication of malaria vectors. *Anopheles pseudowillmori* and *Anopheles culicifacies* are suspected to be the main vectors in Bhutan [11]. The porous borders with the malaria-endemic Indian states of Assam and West Bengal permit easy movement of people between the two countries for employment opportunities and business, presenting a high risk of malaria importation into Bhutan [18].

As Bhutan embarks on the path to malaria elimination, the key focus of the malaria programme includes ensuring full population coverage of preventive measures such as long-lasting insecticidal bed nets (LLINs) and indoor residual spraying (IRS), and access to treatment in target areas. The defining aspects of malaria elimination programmes are: detection of all malaria cases, prevention of onward transmission, management of malaria foci and management of importation of malaria parasites. Elimination needs a relentless focus on surveillance and response and especially on the identification and rapid elimination of foci of infections, both symptomatic and asymptomatic [19]. The malaria surveillance system currently used in Bhutan involves passive reporting of fever and malaria cases and it is not designed to detect asymptomatic cases, which are important contributors to transmission and potential resurgence. There is a need in elimination programmes for the identification of foci of parasite transmission through active surveillance. There is also a need to focus on preventing importation of malaria through proactive case detection at borders, screening of high-risk migrants and the implementation of cross-border initiatives [6, 20, 21].

A primary front-line malaria prevention strategy in Bhutan includes the mass distribution of LLIN in the endemic districts of the country. Between 2006 and 2010, the Vector-borne Disease Control Programme (VDCP) under the Department of Public Health (DoPH) of the Ministry of Health (MoH) of Bhutan, distributed over 228,053 LLINs in these districts, supported by grants from the Global Fund to Fight AIDS, Tuberculosis and Malaria (GFATM) [11]. The success of LLINs as a means of eliminating malaria depends on the willingness of the people to use the LLINs regularly. Maintaining coverage and use of LLINs, preventing importation of malaria from India, and the presence of possible reservoirs among people with asymptomatic infections, are the major challenges to malaria elimination in Bhutan.

This study aimed to assess the coverage, use and ownership of LLINs and factors associated with LLIN use in four selected sub-districts of Sarpang and Samdrup Jongkhar, two historically high-incidence districts of Bhutan on the border with India. Additional aims were to quantify the prevalence of asymptomatic infection with *Plasmodium falciparum* and *Plasmodium vivax* infection in the four sub-districts at a single time point during the peak malaria season, and to assess Bhutan’s progress towards malaria elimination.

## Methods

### Definitions

Definitions for several terms used in this study are provided below:

Household (HH): a unit headed by a male or female with his/her dependents and spouse, and who share a cooking pot/common eating place and sleep under one roof.

LLIN: nets that were distributed by the VDCP, which had deltametherin impregnated in the fibers of the net and which do not need additional impregnation throughout the entire four-year life span of the net.

Regular use of LLINs: all members of the HH sleep under LLINs, including guests, throughout the year.

LLIN ownership: HH having the LLINs distributed by VDCP.

Asymptomatic malaria: individuals returning a positive malaria diagnostic test result but not presenting with any of the classical symptoms such as fever, chills and rigor, sweats, headaches, nausea and vomiting, body aches and malaise.

### Study area and participant recruitment

**Figure 2:**
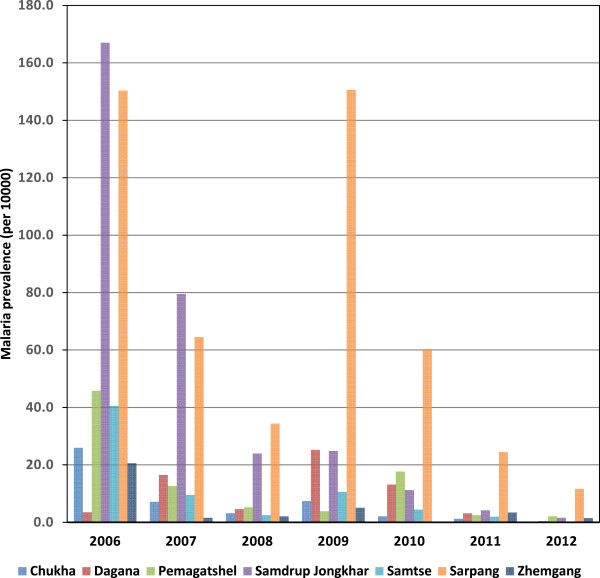
**Malaria trend (incidence) in seven endemic districts of Bhutan from 2006–2012.** (Source: Malaria cases VDCP, Department of Public Health, Ministry of Health; Population of districts from National Statistical Bureau, Bhutan).

Samdrup Jongkhar and Sarpang districts were selected for the study because these districts have persistently had the highest incidence of cases of malaria in Bhutan over the last seven years (Figure 2). The rest of the districts did not report any, or reported very few cases in the last few years. Of note, even the highest-incidence areas of Bhutan are classified as low-endemicity areas, so the highest incidence areas are also likely to be those with the highest prevalence of asymptomatic infections (unlike the scenario in many highly endemic, stable-transmission areas of the world). Two sub-districts were selected from each district on the basis of them having the highest numbers of malaria cases in their respective district. Hence the study specifically targeted areas where malaria was most commonly reported. Attempts were made to survey every HH within the selected sub-districts. Any HH that was unattended on the day of interview was not included in the study. A single respondent, usually the head of the HH, was selected to complete a personal interview with a member of the study team. However, if the HH head was absent on the day of interview, the next eldest person was selected. During the interview, respondents were administered a pretested, structured questionnaire on household LLIN ownership and use.

In addition to the HH survey, a sample of residents was asked to provide a blood sample for malaria diagnosis. To select this sample random household selection was conducted from a geographical reconnaissance (GR) dataset housed in a spatial decision support system (SDSS) that uses the geographical information system (GIS) *Quantum* GIS (QGIS) as its platform. The "Research Tools - Random Selection" geo-processing application within QGIS was used to randomly select 25 HH located within each selected sub-district from the GR dataset. Within each selected HH, two adults and two children (<12 years of age) were selected. The inclusion criteria were: (1) residing in the locality for at least eight weeks prior to the date of testing; and, (2) willingness to undergo the blood test after signing the informed consent form or consent being obtained from parents or guardians of the children. Exclusion criteria were: (1) suffering from other diagnosed co-morbidities; (2) pregnancy; and (3) received/receiving treatment for either *P. falciparum* or *P. vivax* infection during the last eight weeks. Each participant provided a blood sample for malaria diagnosis using the First Sign Para-View 2 rapid diagnostic test (RDT) (Diagnova, Division of RFCL Limited, India).

### Data collection

The survey was carried out in August 2013, coinciding with the historical peak of the malaria transmission season. Based on logistical criteria, blood samples for malaria diagnosis were to be collected from 400 individuals from 25 HH each in four sub-districts and four participants from each HH. The questionnaire used in the HH survey contained questions relating to: (1) characteristics of the respondent (age, gender, whether the respondent was the head of the HH, and occupation); (2) the number of HH members and their age and sex; (3) indicators of socio-economic status and wealth of the HH such as house type, income and ownership of assets (television, refrigerator, electric rice cooker and curry cookers, car, power tiller, rice mill, power chain and bicycle); and (4) ownership and regular use of LLINs based on a measure of individual use.

### Statistical analysis

Data entry was done in Microsoft Excel and analysis was carried out using the statistical package STATA 12.1 (Stata Corporation, College Station, Texas, USA). The primary outcomes of interest were LLIN ownership, LLIN usage and use of LLINs for purposes other than protection against the bite of mosquitoes. The study aimed to determine the frequency and distribution of socio-economic characteristics of the HH surveyed and potential factors associated with LLIN ownership and usage.

Principal component analysis (PCA) was used to derive a socio-economic index based on the types of house and ownership of HH items such as television, refrigerator, electric rice cooker and curry cooker, car, power tiller, rice mill, power chain and bicycle. Using the factor scores from the first principal component as weights, a HH socio-economic score variable was constructed. The scores were used to classify the HH into five broad socio-economic quintiles: least poor, less poor, poor, more poor and most poor.

Bivariate and multivariable logistic regression models for LLINs use and use of LLINs for purposes other than malaria prevention were built using backward elimination to identify significant covariates. An alpha level of 0.10 was used to determine which variables remained in the model. A value of p ≤ 0.05 was considered significant. All explanatory variables in the multivariable model were tested to ensure there was no multi-collinearity in the final model.

### Ethical clearance

Ethical approval for this study was provided by the Research Ethics Board of Health (REBH), MoH, Royal Government of Bhutan (reference number: REBH/Approval/2013/014) and the Human Research Ethics Committee of the University of Queensland (reference number: 2013000884). Verbal permission from local community leaders was sought prior to conducting the survey and examination of blood using RDTs. Written informed consent was obtained from the head of each HH or questionnaire respondent. Interviewers explained the general purpose, benefits, and any risks of the survey to each respondent in his or her local language, and respondents had the right to refuse participation in the survey at any point. Written consent for the participants undergoing the blood test was obtained. For child participants, consent for the testing of blood was obtained from a parent or guardian.

## Results

### Result of blood test for malaria infection using rapid diagnostic test

Malaria diagnosis using the RDT returned valid results for 380 individuals. Children (≤12 years) made up 48.9% (186) of participants while 41.6% (158) were male. All the RDTs were negative for malaria parasites, including either *P. falciparum* or *P. vivax*. Post-hoc analysis, using an exact hypothesis test for a binomial proportion when the proportion is low, indicates that having achieved a sample size of 380 and zero positives, this showed that the prevalence of asymptomatic infection in the population was statistically significantly less than 1% (two-sided test for prevalence <1%, p = 0.037; 95% binomial exact CI for the observed prevalence 0-0.0097). This provided a satisfactory degree of precision to establish a very low prevalence of malaria infection in the population.

### Demographic characteristics of respondents

Out of a total of 1,322 HH in the four subdistricts (Chuzergang 360, Langchenphu 302, Phuntshothang 359 and Umling 301), 1,223 HH (92.5% of total HHs) were administered the questionnaire. The numbers of HH included in each sub-district were: Langchenphu 23.8% (291); Phuntshothang 26.2% (320); Chuzergang 27.0% (330); and, Umling 23.1% (282). Almost 70% (846) of the 1,223 interviewees were heads of HH, and 52.0% (635) were female. The median age of respondents was 42 years (range 14-89 years). The most frequent occupation of the respondents was farming (77.3%, 942 respondents), followed by civil service (9.4%, 115 respondents). Eighty-five per cent of the interviewees (1,040) were married, whereas 8.8% (108) were single.

### Socio-demographic characteristics of households

The total population represented by the HH survey was 5,379 with females making up 51.4% (2,767) of the sample. Children aged < five years comprised 10.3% (555) of the represented population (Table 1). The average number of occupants per HH was 4.4 (range 1-12). The most frequent category of HH income was < Nu 1,500 per month (US$1 = Nu 59.55) (38.9%, 474 respondents), followed by Nu 1,501-3,000 (27.2%, 331 respondents). Only 8.9% (108) of HH had an income > Nu 10,000 per month. The most frequent housing construction type was brick and cement (38.5%, 470 respondents), followed by wood and mud (29.7%, 363 respondents). For ownership of HH items indicative of socio-economic status, the most common item was an electric rice cooker (89.3%, 1,090 respondents), followed by an electric curry cooker (79.7%, 973 respondents). Fifty-nine per cent (724) of the HH owned a television and 51.2% (625) of HH owned a refrigerator. Three per cent (40) of HH owned other items such as a car, rice mill, tractor, or power chain. A majority of the HH (70.2%; 856) were located within one hour walking distance and 27.3% (333) of HH were located one to three hours’ walking distance from the health centre (Table 1).

**Table 1:** **Attribute of household and characteristics of long-lasting insecticide-treated net ownership and use in four sub-districts in Bhutan, 2013**

Attribute	Number	%
Male	2,612	48.6
Female	2,767	51.4
Children <5 years	555	10.3
Children 6-12 years	902	16.8
Young adults 13-24 years	1,090	20.3
Adults >25 years	2,831	52.6
**Income** ^*^		
<Nu 1,500	474	38.9
Nu 1,501-3,000	331	27.2
Nu 3,001-5,000	184	15.1
Nu 5,001-10,000	122	10.0
>Nu 10,000	108	8.9
**Ownership of household items**		
Television	724	59.3
Refrigerator	625	51.2
Rice cooker	1,090	89.3
Curry cooker	973	79.7
Boiler	167	13.7
Other things	40	3.3
**Types of house**		
Hut^**^	223	18.3
Wood and mud	363	29.7
Stone and wood	166	13.6
Bricks and cement	470	38.5
**Socio-economic quintile of household**		
Most poor	282	23.1
More poor	208	17.1
Poor	297	24.3
Less poor	373	30.6
Least poor	60	4.9
**LLINs owned by household**		
Yes	1,203	99.0
No	12	1.0
**Members of households sleeping regularly under LLINs**		
Yes	1,145	93.9
No	75	6.1
**Period when LLINs were not used**		
Summer months	10	14.7
Both summer and winter months	4	5.9
Winter months	53	77.9
Others	1	1.5
**Respondents slept under LLINs the night before the survey**		
Yes	1,190	98.4
No	20	1.7
**Frequency of LLIN washing**		
<6 months	27	2.2
Every six months	806	67.0
7-8 months	15	1.3
>9 months	164	13.6
Never	191	15.9
**Action taken in case net was torn**		
Sleep without bed nets	5	0.4
Repair the bed nets	1,135	94.3
Buy a new bed net	32	2.7
Do not know	23	1.9
**Hanging of LLINs kept during day**		
Hang in the sleeping place	1,161	96.6
Keep in cardboard or box	36	3.0
Keep in other place	4	0.3
**Location of households from the nearest health centre**		
<1 h walking distance	856	70.2
1-3 h walking distance	333	27.3
>3 h walking distance	31	2.5

*US$ 1 = Nu 59.55.

^**^made of bamboo which can be woven or smashed bamboo.

### Long-lasting insecticide-treated nets coverage and use

A high coverage of LLINs was reported among the surveyed HH, with 99.0% (1,203) of HH having LLINs. Most people within the HH (93.9%; 1,145) reported they regularly slept under LLINs, and 98.4% (1,190) of respondents slept under LLINs the night before the survey. Among the respondents who reported that they did not always sleep under LLINs (75 HH), 77.9% (53) said they stopped sleeping under LLINs during the winter months. LLINs were washed every six months in 67.0% (806) of HH while 15.9% (191), never washed. In the event of a net being torn, 94.3% (1,135) reported that they would repair the net and 2.7% (32) reported that they would buy a new net. Most respondents (96.6%) reported that they kept the LLINs hanging in the sleeping area during the day (Table 1).

### Factors associated with long-lasting insecticide-treated net use

The HH that washed LLINs more frequently than every six months (OR = 0.2, <0.0001, AOR = 0.2, p = 0.026), less frequently than every nine months (OR = 0.2, p < 0.0001; AOR = 0.1, p < 0.0001) and that never washed LLINs (OR = 0.5, p = 0.03; AOR = 0.5, p = 0.10) were less likely to sleep under LLINs compared to HH that washed their nets as per manufacturer instructions (every six months) (Table 2).

**Table 2:** **Factors associated with use of long-lasting insecticide-treated nets in Bhutan, 2013**

Net use	Unadjusted		Adjusted	
	Odds ratio	P value	Odds ratio	P value
	(95% CI)		( 95% CI)	
**Washing of LLINs (1,172)**				
Every 6 months (801)	Ref			
<6 months (26)	0.2 (0.1, 0.4)	<0.0001*	0.2 (0.1, 0.8)	0.026*
>9 months (164)	0.2 (0.1, 0.4)	<0.0001*	0.1 (0.1,0.3)	<0.0001*
Never washed (191)	0.5 (0.2, 0.7)	0.03*	0.5 (0.2, 1.1)	0.10
**Socio-economic quintile (1,200)**				
Most poor (278)	Ref		Ref	
More poor (205)	1.0 (0.4, 2.4)	0.97	0.8 (0.3, 2.2)	0.65
Poor (295)	0.7 (0.3, 1.5)	0.34	0.5 (0.2, 1.2)	0.13
Less poor (363)	1.1 (0.5, 2.3)	0.91	0.9 (0.3, 2.7)	0.87
Least poor (59)	0.1 (0.1, 0.3)	<0.0001*	0.2 (0.1, 0.5)	0.002*
**Household members (1,189)**				
< 3 members (419)	Ref		Ref	
4-6 members (610)	1.1 (0.7, 1.9)	0.66	1.0 (0.5, 1.9)	0.98
7-9 members (169)	1.9 (0.8, 4.8)	0.15	2.5 (0.8, 7.7)	0.11
**Household income per month (1,199)**				
< Nu 1,500 (472)	Ref		Ref	
Nu 1,501-3,000 (327)	1.5 (0.7, 3.2)	0.32	0.8 (0.3, 2.0)	0.66
Nu 3,001-5,000 (180)	4.1 (1.0, 17.9)	0.06	2.2 (0.5, 10.6)	0.33
Nu 5,001-10,000 (117)	0.4 (0.2, 0.8)	0.007*	0.3 (0.1, 0.9)	0.027*
>Nu 10,000 (103)	0.2 (0.1, 0.3)	<0.0001*	0.1 (0.04, 0.3)	<0.0001*
**Location of households from the nearest health centre (1,200)**				
<1 hrs (840)	Ref		Ref	
1–3 hrs (329)	0.5 (0.3, 0.9)	0.012*	0.3 (0.1, 0.7)	0.002*
>3 hrs (31)	1			
**Action taken if LLINs are torn (1,182)**				
Repair the LLINs (1,122)	Ref		Ref	
Do not know (22)	0.1 (0.1 0.3)	<0.0001*	0.1 (0.03, 0.3)	<0.0001*
Buy new one (38)	0.2 (0.1, 0.3)	<0.0001*	0.5 (0.2, 1.5)	0.24
**Hanging of LLIN during day (1,184)**				
Hang in sleeping area (1,147)	Ref		Ref	
Keep in the box (33)	0.1 (0.04, 0.2)	<0.0001*	0.1 (0.1, 0.4)	<0.0001*
Other place (4)	0.1 (0.02, 1.4)	0.09	0.3 (0.02, 3.8)	0.33

Unadjusted odds ratio (OR) was obtained from bivariate logistic regression and adjusted odds ratio (AOR) was obtained from multivariable logistic regression.

*significant at p < 0.05.

The respondents of HH in the least poor socio-economic quintile were less likely to sleep under a LLIN (OR = 0.1, p < 0.0001; AOR = 0.2 p = 0.002) compared to the poorest quintile. Similar results were obtained when income was used as an explanatory variable: respondents of HH with an income of Nu 5,001-10,000 (OR = 0.4, p = 0.007; AOR = 0.3, p = 0.027) and Nu >10,000 (OR = 0.2, p < 0.0001; AOR = 0.1, p < 0.0001) were less likely to use LLINs as compared to HH with an income of Nu <1,500.

Household located one to three hours walking distance from the nearest health centre were less likely to use LLINs compared to HH located < one hours walking distance (OR = 0.5, p = 0.012 AOR = 0.3, p = 0.002). In the event of LLINs being torn, HH where the respondent reported that they did not know what to do (OR = 0.1, p < 0.0001; AOR = 0.1, p < 0.0001) and who reported that they would buy new nets (OR = 0.2, p < 0.0001) were less likely to sleep under LLINs as compared to HH who said they would repair torn LLINs. The HH who kept their LLINs in a box were less like to sleep under LLINs (OR = 0.1, p < 0.0001; AOR = 0.1, p < 0.000) compared to those who hung the LLIN in the sleeping area during the day (Table 2).

### Use of long-lasting insecticide-treated nets for non-intended purposes

It was reported that LLINs were used for purposes other than malaria prevention by 4.3% (50) of HH. The HH in the poor and less poor socio-economic quintiles were less likely to use LLINs for non-intended purposes compared to the poorest quintile (OR = 0.4, p = 0.018 and OR = 0.1, p < 0.0001), respectively. However, after adjusting for other variables, the associations were not significant (AOR = 0.9, p = 0.70 and AOR = 0.3, p = 0.09, respectively). The HH located one to three hours’ walking distance from the nearest health centre were more likely to use LLINs for non-intended purposes (OR = 8.8, p < 0.0001 and AOR = 10.4, p < 0.0001, respectively) than HH located < one hours’ walking distance from a health centre. Incomes of HH, number of HH members, action taken in case of LLINs being torn and hanging of LLINs during the day in different locations were not statistically associated with use of LLINs for non-intended purposes (Table 3).

**Table 3:** **Factors associated with use of long-lasting insecticide-treated nets for non-intended purposes in Bhutan, 2013**

Net used for other purpose	Unadjusted		Adjusted	
	Odds ratio	P value	Odds ratio	P value
	(95% CI)		(95% CI)	
**Wealth quintile (1,200)**				
Most poor (278)	Ref		Ref	
More poor (205)	0.7 (0.3, 1.4)	0.26	0.8 (0.3, 1.9)	0.61
Poor (295)	0.4 (0.2, 0.9)	0.018*	0.9 (0.4, 1.2)	0.70
Less poor (363)	0.1 (0.03, 0.3)	<0.0001*	0.3 (0.1, 1.2)	0.09
Least poor (59)	0.2 (0.02, 1.4)	0.1	1.0 (0.1, 8.8)	0.98
**Household members (1,189)**				
< 3 members (419)	Ref		Ref	
4-6 members (610)	1.0 (0.6, 1.8)	1.0	1.2 (0.6, 2.4)	0.67
7-9 members (169)	0.4 (0.1, 1.2)	0.1	0.4 (0.1, 1.5)	0.17
**Household income per month (1,199)**				
< Nu 1,500 (472)	Ref		Ref	
Nu 1,501-3,000 (327)	0.9 (0.5, 1.6)	0.64	3.2 (1.5, 7.1)	0.003*
Nu 3,001-5,000 (180)	0.6 (0.2, 1.4)	0.23	2.2 (0.7, 6.7)	0.17
Nu 5,001-10,000 (117)	0.1 (0.02, 1.1)	0.06	0.5 (0.1, 4.1)	0.53
>Nu 10,000 (103)	0.2 (0.02, 1.3)	0.09	1.4 (0.1, 13.1)	0.79
**Location of households from the nearest health centre (1,169)**				
<1 hrs (840)	Ref		Ref	
1–3 hrs (329)	8.8 (4.3, 18.2)	<0.0001*	10.4 (4.5, 24.1)	<0.0001*
**Action taken if LLINs are torn (1,182)**				
Repair the LLINs (1,122)	Ref		Ref	
Do not know (22)	1.1 (0.2, 8.4)	0.92	1.5 (0.2, 12.4)	0.71
Buy new one (38)	1.3 (0.3, 5.4)	0. 75	0.8 (0.1, 6.5)	0.80
**Keeping LLIN during day (1,184)**				
Hang in sleeping area (1,147)	Ref		Ref	
Keep in the box (33)	1.6 (0.4, 6.8)	0.53	1.8 (0.4, 9.2)	0.48

Unadjusted odds ratio (OR) was obtained from bivariate logistic regression and adjusted odds ratio (AOR) was obtained from multivariable logistic regression.

*significant at p < 0.05.

## Discussion

This study focused on LLIN coverage and use in areas of Bhutan that traditionally had the highest incidence of reported malaria. In these areas, numbers of malaria cases reported through passive case detection has continually decreased. However, little is known about asymptomatic malaria since active case detection has not been undertaken. As part of this study, 380 participants provided blood samples to reveal a zero prevalence of asymptomatic malaria, which is encouraging for malaria elimination efforts. However, a larger sample would be required to provide clear evidence of cessation of malaria transmission.

This study found a very high coverage of LLINs in four sub-districts of Bhutan. The VDCP strategy of distributing free LLINs to achieve a target of universal coverage in the malaria endemic districts of Bhutan appears to have worked well. The previous mass distribution of LLINs in the study sub-districts was carried out in 2010 and the most recent round of mass distribution of LLINs was carried out in December 2013, soon after the current study was conducted, which is likely to further enhance LLIN coverage in the malaria-endemic districts of Bhutan. A high coverage of LLINs with consistent use of LLINs throughout the year is important to prevent and protect the population from malaria infection and to achieve elimination by 2016, which is the stated national goal of Bhutan.

The percentage of HH sleeping under LLINs regularly was found to be 93.9%, with the reported percentage dropping during the winter months. As reported in other studies, the main reason for not sleeping under LLINs was the perception that there were no mosquitoes during the winter months [22]. Although no malaria infections were detected in this study, importation is a constant threat so there is a need to sensitize the community to the importance of LLIN adherence throughout the year, with emphasis on the risk of malaria transmission occurring year-round. This may require routine HH visits by trained community health workers, or providing education during the mass distributions of LLINs, mass IRS rounds, or regular dedicated malaria awareness campaigns.

LLIN maintenance is an important issue for malaria elimination. Even though 67% of the respondents washed their net regularly (at least once every six months), almost 16% never washed their LLINs. Washing at regular time intervals is important because dirt and other particles on the LLINs may act as a barrier, reducing the effectiveness of the chemicals on the net. The respondents who washed LLINs very frequently (<six months), less frequently (>nine months) and who never washed were less likely to sleep under LLINs as compared to respondents that washed LLINs as per the manufacturers’ guidelines (every six months). This might reflect that a stronger commitment to use LLINs is accompanied by a commitment to maintain them. Most of the respondents (94.27%) said they would repair nets if they were torn. The repair of minor tears of LLINs can help increase the effective lifespan of LLINs. Washing of LLINs and repair of LLINs are important indicators of the care and maintenance of LLINs. Hanging LLINs during the day has been identified as a factor strongly associated with LLIN use [23, 24]. Most of respondents, 96.6% hung their LLINs in the sleeping area during the day time. This supports the assessment that the use of LLINs in the study area was high. Other benefits of keeping the net hanging include that chemicals on the LLINs will deter mosquitoes from coming into the rooms, having an additional preventive effect on biting [25, 26].

HH in the least poor socio-economic quintile were less likely than the poorest HH to use LLINs, and similar findings were reported in other studies [27–29]. The houses in the higher socio-economic quintiles were better constructed, with a likely perception of mosquitoes being less able to enter the house. These HH could be using other protective measures such as mosquito repellents or installation of screens on windows and doors; however this information was not collected during the study. Households located one to three hours’ walking distance from the nearest health centre were less likely to use LLINs compared to HH located one hour from the health centre, possibly because HH that were nearer to the health centres are better informed on the risks of getting malaria if LLINs were not used regularly. Similar findings have been made in other studies [30].

It has been reported that mosquito nets have been used for purposes other than malaria protection, including fencing gardens, storing grains, drying and as fishing nets [22, 23, 31]. It has also been suggested that this is the case in the endemic districts of Bhutan. However, reported use of LLINs for other purposes in the study was low, as has been found elsewhere [32], most likely reflecting a high degree of understanding of the importance of LLINs in preventing malaria.

There are some potential limitations to the current study which should be considered. Firstly, LLINs ownership and use by HH were based on self-report without verification. Secondly, the respondents may have over-reported net use, or under-reported the use of LLINs for alternate purposes, on the basis of social desirability, especially given that the interview was conducted by the malaria technicians of the health centers of the catchment area. In terms of using RDTs for malaria diagnosis, while the sensitivity and specificity of the RDT are reported to be high [33], however reduced sensitivity might occur with low parasite densities and exposure of the RDT to extreme temperatures [34–37].

## Conclusions

A zero prevalence of asymptomatic malaria and a high coverage of LLINs was reported in the study area with regular use throughout the year. The use of LLINs for non-intended purposes was low. Never-the-less, there is a need to educate the small proportion of people not sleeping under LLINs, particularly in the winter months, to use LLINs throughout the year, and to promote regular washing of LLINs among 16% of respondents who never wash their LLINs. Based on the findings of the current study, it appears that Bhutan is on course to achieve malaria elimination.
